# Repeated horizontal transfers of four DNA transposons in invertebrates and bats

**DOI:** 10.1186/s13100-014-0033-1

**Published:** 2015-01-17

**Authors:** Zhou Tang, Hua-Hao Zhang, Ke Huang, Xiao-Gu Zhang, Min-Jin Han, Ze Zhang

**Affiliations:** School of Life Sciences, Chongqing University, Chongqing, 400044 China; College of Pharmacy and Life Science, Jiujiang University, Jiujiang, 332000 China; College of Forestry and Life Science, Chongqing University of Sciences and Arts, Yongchuan, Chongqing, 40216 China

**Keywords:** Horizontal transfer, *CACTA* transposons, Mammals, Recent activity

## Abstract

**Background:**

Horizontal transfer (HT) of transposable elements (TEs) into a new genome is considered as an important force to drive genome variation and biological innovation. However, most of the HT of DNA transposons previously described occurred between closely related species or insects.

**Results:**

In this study, we carried out a detailed analysis of four DNA transposons, which were found in the first sequenced twisted-wing parasite, *Mengenilla moldrzyki*. Through the homology-based strategy, these transposons were also identified in other insects, freshwater planarian, hydrozoans, and bats. The phylogenetic distribution of these transposons was discontinuous, and they showed extremely high sequence identities (>87%) over their entire length in spite of their hosts diverging more than 300 million years ago (Mya). Additionally, phylogenies and comparisons of transposons versus orthologous gene identities demonstrated that these transposons have transferred into their hosts by independent HTs.

**Conclusions:**

Here, we provided the first documented example of HT of *CACTA* transposons, which have been so far extensively studied in plants. Our results demonstrated that bats had continuously acquired new DNA elements via HT. This implies that predation on a large quantity of insects might increase bat exposure to HT. In addition, parasite-host interaction might facilitate exchanging of their genetic materials.

**Electronic supplementary material:**

The online version of this article (doi:10.1186/s13100-014-0033-1) contains supplementary material, which is available to authorized users.

## Background

DNA-mediated or class 2 transposons were one class of transposable elements (TEs). Most DNA transposons transpose via a ‘cut and paste’ mechanism implemented by transposases. They were generally characterized by terminal inverted repeats (TIRs) and target site duplication (TSD) [1]. Based on their transposases, DNA transposons could be classified into 19 superfamilies, including *Tc1/mariner*, *hAT*, *PiggyBac*, *CACTA*, *MuDR*, *Merlin*, *Transib*, *P*, *PIF/Harbinger*, *Mirage*, *Zator*, *Ginger*, *Kolobok*, *Chapaev*, *Novosib*, *Rehavkus*, *PHIS*, *Sola*, and *Academ* [2,3].

Although the possibility of stochastic loss suggests that TEs should be a seemingly inevitable vertical extinction in their original host genomes, TEs are widespread in organisms [1,4-6]. Horizontal transfer (HT) is a process of genetic material exchanging among non-mating species or isolated species. HT of a transposon into a new genome allows the element to evade inevitable extinction, suggesting that HT plays important roles in the persistence of TEs [4]. In addition, HT of TEs into a new genome is also regarded as important forces to drive genome variation and biological innovation.

Generally, there are three criteria used to infer HT events: (1) high sequence similarity of TEs from divergent taxa, (2) incongruence between TE and host phylogeny, and (3) a patchy TE distribution within a group of taxa [7,8]. The first documented example of HT of TEs was the *P* element of *Drosophila* [9]. More than 330 cases (188 cases for DNA transposons and 142 cases for RNA transposons) of eukaryote-to-eukaryote HT events of TEs were described so far [10]. However, no documented example of HT has been described for the *CACTA* superfamily of DNA transposons, which so far has extensively been studied in plants [6,11]. In addition, most of HT of DNA transposons (122 out of 188) previously described occurred between closely related species or insects [10].

In this study, we described four DNA transposons which were present in diverse invertebrate and vertebrate animals. The combination of high identity levels between TEs despite deep divergence times of their host taxa, patchy TE taxonomic distribution, and lower genetic distances for TEs than for host genes clearly demonstrated that they had horizontally transferred into their hosts.

## Results

### Distribution patterns of four DNA transposons

**Figure 1:**
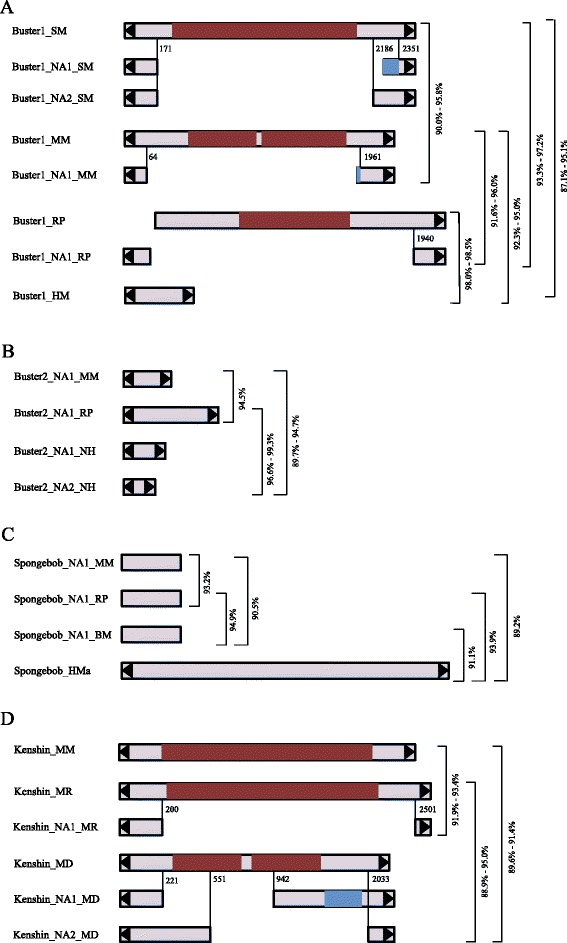
**Diagram showing the detail information about transposons of** ***Buster1*** **(A),** ***Buster2*** **(B),** ***Spongebob*** **(C), and** ***Kenshin*** **(D).** Black triangles represent the TIRs. Gray rectangles represent non-coding regions, and purple rectangles indicate transposase regions. Percentages of identity were calculated using Bioedit. Blue regions represent the variable area of transposons.

**Figure 2:**
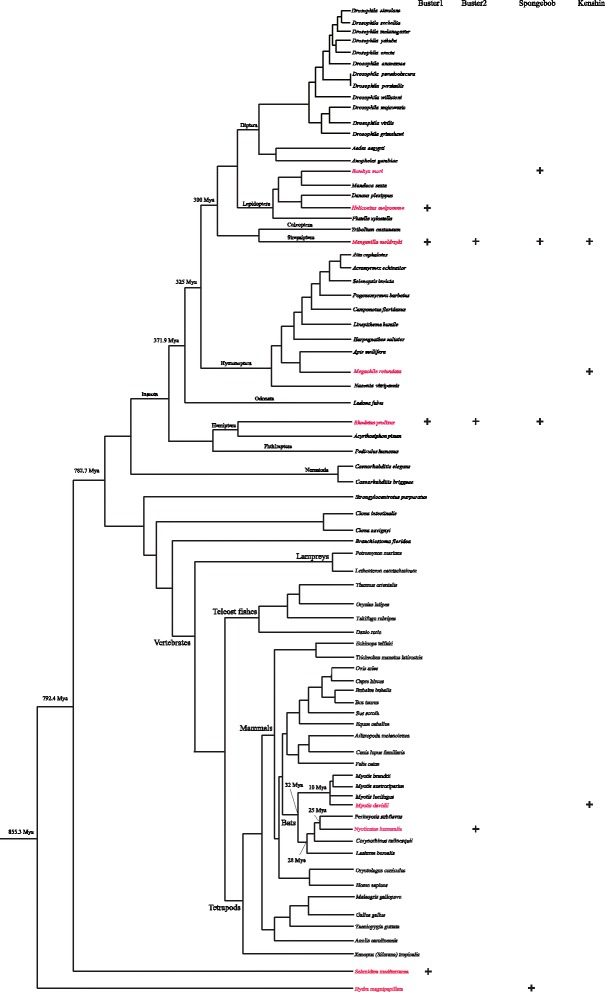
**The taxonomic distribution of** ***Buster1*** **,** ***Buster2*** **,** ***Spongebob*** **, and** ***Kenshin*** **among species for which genome sequences are available.** Presence of these transposon families in each lineage are denoted by plus sign. Species divergence is taken from previous literatures [14-17].

The twisted-wing parasite, *Mengenilla moldrzyki*, is the first sequenced species of Strepsiptera [12]. Nineteen seventy potential TEs of the twisted-wing parasite were downloaded from Dryad Digital Repository (http://datadryad.org/resource/doi:10.5061/dryad.ts058.2). The screening of the distribution of these transposons revealed that four of these 1970 TEs yielded highly significant (>87%) hits in many diverse species, not only in insects but also in freshwater planarian, hydrozoans, or bats (Table 1, Figures 1 and 2). These four DNA transposons formed the start pointing of this study. They were grouped into *hAT*, *CACTA*, and *piggyBac* superfamilies based on their similarities to known members of these superfamilies (Table 1). Full-length or partial ancestral sequences in each species were reconstructed and compared to each other (Additional file 1: Table S1).

**Table 1:** **Characteristics of four DNA transposons in this study**

**Superfamily**	**Family**	**Organism**	**TEs**	**Length (bp)**	**Copy no.**	**TIRs (5′-3′)**	**Average divergence ± SE**	**References**
*hAT*	*Buster1*	*Schmidtea mediterranea*	*Buster1_SM*	2,463	17	CAGGGCTTCTTAAAC	8.22 ± 5.66	hAT-11_SM[13]
			*Buster1_NA1_SM*	373	270	CAGGGCTTCTTAAAC	1.45 ± 0.65	This study
			*Buster1_NA2_SM*	449	31	CAGGGCTTCTTAAAC	1.45 ± 0.75	This study
		*Mengenilla moldrzyki*	*Buster1_MM*	2,196	3	ND	2.16 ± 3.75	This study
			*Buster1_NA1_MM*	366	44	CAGGCCTTCTTAAACT	9.94 ± 2.83	This study
		*Rhodnius prolixus*	*Buster1_RP*	2,281	3	ND	0.86 ± 1.49	This study
			*Buster1_NA1_RP*	580	17	CAGGGCTTCTTAAACT	1.82 ± 0.85	This study
		*Heliconius melpomene*	*Buster1_NA1_HM*	500	42	CAGGGTTTCTTAAACT	2.32 ± 1.38	nhat-10_Hmel[13]
	*Buster2*	*M. moldrzyki*	*Buster2_NA1_MM*	365	252	CAACGGTGGCCA	13.5 ± 2.16	This study
		*R. prolixus*	*Buster2 _NA1_RP*	908	16	CAGGGGGGGGCCAACCT	4.74 ± 1.51	This study
		*Nycticeius humeralis*	*Buster2 _NA1_NH*	337	>10	CAGGGGTGGCCAACCT	4.81 ± 1.48	nhAT-5a_Nhu[unpublished]
			*Buster2_NA2*_*NH*	246	>104	CAGGGGTGGCCAACCT	4.63 ± 1.87	nhAT-2a_Nhu[unpublished]
*CACTA*	*Spongebob*	*M. moldrzyki*	*Spongebob_NA1_MM*	496	40	ND	9.88 ± 3.95	This study
		*R. prolixus*	*Spongebob_NA1_RP*	433	2	ND	2.74 ± 3.87	This study
		*Bombyx mori*	*Spongebob_NA1_BM*	468	17	ND	10.6 ± 3.69	This study
		*Hydra magnipapillata*	*Spongebob_ HMa*	5,836	441	CCCAGCCAACATTGAC (17)	5.94 ± 3.48	EnSpm-4N1_HM[2]
*piggyBac*	*Kenshin*	*M. moldrzyki*	*Kenshin_MM*	2,244	15	CACTAGA	13.2 ± 5.49	This study
		*Megachile rotundata*	*Kenshin_MR*	2,520	9	CACTAGA	9.91 ± 3.84	This study
			*Kenshin_NA1_MR*	235	46	CACTAGA	2.82 ± 2.24	This study
		*Myotis davidii*	*Kenshin_MD*	2,108	1	CACTAG	ND	This study
			*Kenshin_NA1_MD*	1,267	23	CACTAGA	2.16 ± 0.60	This study
			*Kenshin_NA2_MD*	627	3	CACTAGA	2.46 ± 0.20	This study

**Figure 3:**
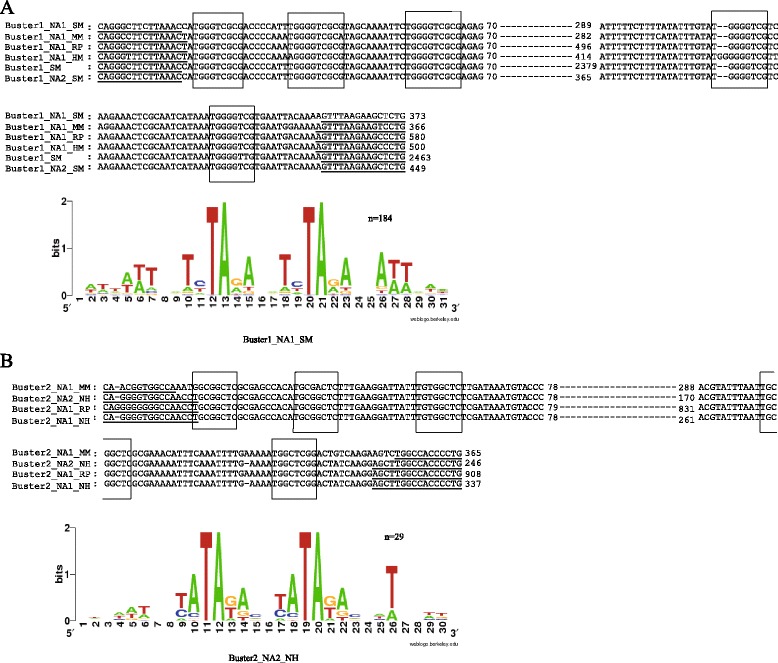
**Structure characteristics of** ***Buster1*** **(A) and** ***Buster2*** **(B) and sequence logo of the regions flanking** ***Buster1_NA1_SM*** **and** ***Buster2_NA2_NH*** **insertions.** The 15 nt upstream and downstream of all full-length copies of these families in *Schmidtea mediterranea* and *Nycticeius humeralis* are presented in each logo. The vertical axis is a measure of sequence information, which has a maximum value of 2 and is proportional to the level of sequence conservation at each position. The rectangles indicate their direct repeats. Their TIRs were shown using underlines, and numbers indicated their alignment positions.

**Figure 4:**
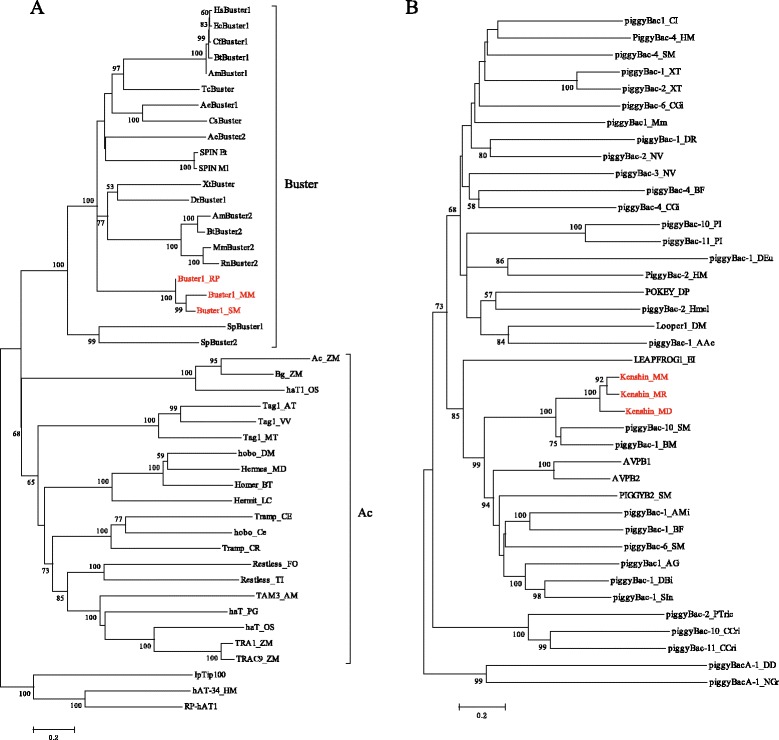
**Phylogenetic trees of** ***Buster1*** **(A) and** ***Kenshin*** **(B).** *Buster1* and *Kenshin* identified in this study were shown in red. Representatives of transposons of the *hAT* superfamily were obtained from previous studies [20,21]. Representatives of *piggyBac* transposons were downloaded from Repbase [2]. Bootstrap value <50% was not shown.

The first DNA transposon, called *Buster1*, was found in *M. moldrzyki*, *Schmidtea mediterranea*, *Rhodnius prolixus*, and *Heliconius melpomene* (Table 1 and Figure 2). Except for *H. melpomene*, this element in other three species generated both autonomous and non-autonomous elements (including miniature inverted-repeat transposable elements (MITEs)). Multiple alignments of MITEs and its autonomous ancestors indicated that they originated by internal deletions from master elements. These results supported the hypothesis that MITEs borrowed the machinery of autonomous DNA transposons to transpose [1]. Insertion bias and phylogenetic analysis demonstrated that they belonged to one member of the *Buster* family of the *hAT* superfamily (Figures 3A and 4A). Structure analysis indicated that the subterminal regions of the elements contain TGGGTCGCG tandem repeats. Generally, short repeats in subterminal regions have been used to distinguish different *hAT* transposons [18]. Thus, *Buster1* might represent a novel member of the *Buster* family. Moreover, the repetitive motif identified in *Buster1* might have important structural or functional roles during their transposition [19]. These elements identified in these hosts which diverged more than 300 million years ago (Mya) [14] revealed high nucleotide sequence identity (>87%) over almost the full length (Figure 1A), suggesting that these elements were derived from the same active ancestral element. *Buster1* was found in low copy number (<50) in most species, except for the freshwater planarian *Schmidtea mediterranea* (*Buster1_NA1_SM*) where this element was found more than 250 copies (Table 1). The average sequence divergence between *Buster1_NA1_SM* copies and its consensus sequence was only 1.45%, indicating that this element might have experienced a burst transposition very recently in the freshwater planarian.

**Figure 5:**
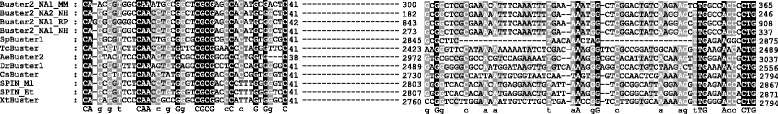
**Multiple alignments of** ***Buster2*** **identified in this study and previously reported** **[** 20 **]** **, showing portions of the highly conserved 5′ and 3′ termini.** Numbers indicated their alignment positions.

The second DNA transposon, called *Buster2*, was found not only in invertebrates (*M. moldrzyki* and *R. prolixus*) but also in one vertebrate (the evening bat *Nycticeius humeralis*) (Table 1 and Figure 2). They have proliferated via amplification of non-autonomous elements in these species. Furthermore, one non-autonomous element was identified in the twisted-wing parasite and triatomine bug, and two non-autonomous elements were identified in the evening bat (Table 1). The successful amplification of these non-autonomous elements was surprising because autonomous partners responsible for their transposition were not found in these species. It is possible that its transposition was catalyzed by different but related autonomous elements of their ancient masters, which was known as cross-mobilization [22]. An alternative explanation is that autonomous elements could have remained polymorphic for a long time in the host population without augmenting its copy number and have been lost through allele sorting. It could also be that the autonomous *Buster2* elements might reside in their host genomes but were not found in this study as a result of incomplete genome sequences. Insertion bias analysis indicated that *Buster2* was also a member of the *Buster* family (Figure 3B). Similar to *Buster1*, *Buster2* elements were flanked by TGCGGCTC tandem repeats. Because *Buster2* elements identified in these species were non-autonomous, we further investigated the similarities of their terminal regions with reported *Buster* elements. Multiple alignments showed that the terminal region of *Buster2* shared high sequence similarities with those of the *Buster* elements (Figure 5), which further demonstrated that it was a *Buster* transposon. Interestingly, similarities of all *Buster* elements were not restricted to their terminal inverted repeats (TIRs) but also extended to about 106 bp of their terminal regions. This also showed that their 5′ terminal regions were more conserved than 3′ terminal regions. These results suggested that these conservative sites in their terminal regions might play important roles during the process of their transposition. This is also consistent with the fact that the *Buster* family might experience a recent burst of amplification based on the phylogeny of their transposases [20].

The third DNA transposon, called *Spongebob*, was found in insects (*M. moldrzyki*, *R. prolixus*, and *Bombyx mori*) and hydrozoans (*Hydra magnipapillata*). Only partial consensus sequences of *Spongebob* could be reconstructed for insects (Table 1 and Figure 1C). In the hydrozoans, *Spongebob* was present in multiple full-length copies (>50), which allowed the reconstruction of a consensus sequence of 5,836 in length. However, it is difficult for us to find the exact transposase encoding by *Spongebob_ HMa* due to stop codons or frameshifts. The first three bases in the TIRs of *Spongebob_ HMa* were CCC, and their copies were flanked by 2 bp target site duplication (TSD) (Additional file 2: Figure S1), suggesting that it was a member of TRC elements of the *CACTA* (also called *En/Spm*) superfamily of DNA transposons [23]. A pairwise comparison of *Spongebob* consensus sequences from the above four species revealed that the elements were more than 89.7% identical over about 430 bp (Figure 1C), suggesting that they should belong to the same family.

The last DNA transposon named as *Kenshin* was shared by the twisted-wing parasite, alfalfa leafcutting bee *Megachile rotundata* and bat *Myotis davidii* (Table 1 and Figure 2). We found one copy of *Kenshin* in the twisted-wing parasite and alfalfa leafcutting bee, which had an intact open reading frame (ORF) encoded a 584- and 581-amino acid (aa) long transposase, respectively. This suggests that the element had an ability of transposition in both species. This element might be also responsible for the amplification of non-autonomous elements in these species. We identified one non-autonomous element in the alfalfa leafcutting bee and two non-autonomous elements in the bat (Table 1). These non-autonomous elements had experienced successful amplification and largely outnumbered their autonomous masters (Table 1). One explanation might be that non-autonomous elements could avoid defense system of their hosts as a result of short sequence length [1]. *Kenshin* elements identified in these species were very similar to each other and diverged by 5.0%–11.1% (Figure 1D). Phylogenetic analysis based on transposases of autonomous elements demonstrated that it was a member of the *piggyBac* superfamily (Figure 4B).

### Evidence for repeated horizontal transfers

Four DNA transposons described here showed extremely high identities (>87%) over the full length at the nucleotide level despite their hosts diverged more than 300 Mya (Figures 1 and 2, respectively). This provided us with convincing evidence to support that these transposons had repeatedly invaded into these species by HTs. However, we should note that these results might result from other evolutionary processes, such as purifying selection acting on transposons or variable rates of the evolution of transposons [24,25]. Therefore, making HT conclusion of these transposons should be cautious.

To obtain more evidence for HTs of these transposons, we investigated the phylogenetic distribution of these transposons. The results indicated that they were discontinuous distribution in species (Table 1 and Figure 2). For example, both *Buster2* and *Kenshin* were only present in two invertebrates and one vertebrate, and they were not identified in all other vertebrate and invertebrate species for which a complete or nearly complete genome is available in the National Center for Biotechnology Information (NCBI) database (>102) [26]. Similar patterns were also observed for *Buster1* and *Spongebob* (Figure 2).

Additionally, in many cases, the sequence identities of these four DNA transposons were extremely high compared with the divergence time of their hosts. For example, there was more than 87% between *Buster2* in the insects and the freshwater planarian, which diverged more than 792 Mya [14] (Figures 1 and 2). Similarly, *Buster2* and *Kenshin* identified in the insects and mammals, which shared the last common ancestor about 782 Mya [14], showed more than 89% identities. Besides, we also found that *Spongebob* in the insects and hydrozoans shared high sequence identities (>89%) at the nucleotide level.

We also observed that phylogenies for *Buster1* and *Buster2* showed a striking lack of structure. For example, phylogenetic analysis based on transposases of *Buster1* showed that this element identified in the twisted-wing parasite was much closer to the freshwater planarian than to another insect, the triatomine bug (Figure 4A). Besides, an unrooted tree based on copies of *Buster2* suggested that *Buster2* elements in the triatomine bug and evening bat were much closer with each other compared with that in the twisted-wing parasite (Additional file 3: Figure S2). All these results were not consistent with vertical inheritance of these transposons.

Finally, our results showed that the nucleotide sequence divergence among four DNA transposons (about 1.5%–13%) was much lower than that observed for three conserved host nuclear genes (about 22%–30%), *heat shock cognate 70*, *Tubulin beta-3*, and *elongation factor 1 alpha*, which were described in our previous study [27]. Therefore, HTs of these transposons might be the only logical explanation for high sequence identities among these transposons in distantly related species.

## Discussion

Here, we performed a detailed analysis of characteristics and evolutionary history of four DNA transposons in diverse species. The combination of high identity levels between TEs despite deep divergence times of their host taxa, patchy TE taxonomic distribution, and lower genetic distances for TEs than for host genes clearly demonstrated that these elements had transferred into these species by independent HTs. We also noted that the phenomenon of HT of *Buster1* had previously been reported [13]. However, the detail information about this transposon remains unknown. In this study, both non-autonomous elements and its autonomous partners were found in the twisted-wing parasite, triatomine bug, and freshwater planarian (Table 1), which would provides us with a better understanding for the evolutionary history of *Buster1*. In addition, structural and phylogenetic analyses showed that *Buster1* was a novel member of the *Buster* family.

Although the distribution of these four DNA transposons in species was patchy, their transfer did not randomly happen since the same species have been independently invaded by different, unrelated TEs but others appear to be immune to HT (Figure 2). For example, three transposons are present in the twisted-wing parasite and triatomine bug, but they are not found in other insects (>30) for which genomic sequences are available. This pattern implies that some taxa might be prone to exchanging of genetic materials or are more hospitable to TEs than others. It is reasonable that species which are vulnerable to HT have a weakened response to TE invasion, which would lead them to lose control of the amplification of the new invader. However, species with a strong resistance would not allow the TEs to amplify in the genome. Similar phenomena have been observed in vertebrates [28].

DNA transposons exist in a wide variety of organisms. However, it was believed that DNA transposons existing in mammals were fossils, and they did not have any ability for mobility in the last 40 Mya [29-31]. This situation has changed when recent DNA transposon activity was discovered in the bats [32-34]. Here, the low average divergence (2.16%–4.81%) between copies of *Buster2* and *Kenshin* and their consensus sequences in the bats strongly suggested that they had been inserted recently (Table 1). Besides, *Buster2* and *Kenshin* were apparently absent from all other mammals (>80) including other seven closely related bats (Figure 2), for which genome sequences are available. Interestingly, *Kenshin* is only present in the genome of the bat *M. davidii* but is not in the other three Myotis genomes sequenced, suggesting that it might be mobilized within the last 10 Mya [15]. These results also implied that bats had continuously acquired new DNA elements via HT. Interestingly, bats belonging to Vespertilionidae family that were the only mammals reported to have recent DNA transposon activity [32-34]. Meanwhile, many of DNA transposons were also horizontally transferred into their hosts [28,35]. However, we should note that HT provides a delivery system for the re-colonization of TEs of genomes and we cannot exclude that DNA transposons might be active in many mammals for which genomes are not sequenced.

Four DNA transposons were found in a wide range of organisms including insects, freshwater planarian, hydrozoans, and bats, suggesting that multiple mechanisms might be involved in their HTs. One interesting finding is the identification of these elements to be present and transferred between insects and bats. The evening bats feed heavily on beetles (Coleoptera), but they also eat moths (Lepidoptera), small flies (Diptera), and other insects [36]. This suggested that predation on a large quantity of insects might increase bat exposure to HT. Another interesting finding is the identification of near identical DNA transposons in insects and the twisted-wing parasite. *M. moldrzyki* is a species of Strepsiptera (Mengenillidae), which infects at least 35 families of insects belonging to seven orders [37]. During the process of parasitism, these parasites obtained nutrients from their hosts [38]. Therefore, parasite-host interaction might facilitate exchanging of their genetic materials.

## Conclusions

In this study, we provided the first documented example of HT of *CACTA* transposons. Our results demonstrated that bats had continuously acquired new DNA elements via HT. This implies that predation on a large quantity of insects might increase bat exposure to HT. In addition, parasite-host interaction might facilitate exchanging of their genetic materials.

## Methods

### Data resources

The silkworm (*Bombyx mori*) assembled genomic sequences were downloaded from Silkworm Genome Database [39] (http://www.silkdb.org/silkdb/). The triatomine bug, *Rhodnius prolixus*, genomic supercontig sequences were downloaded from VectorBase [40] (https://www.vectorbase.org/). Survey sequences from the genomes of five bats (*Myotis austroriparius*, *Lasiurus borealis*, *Corynorhinus rafinesquii*, *Perimyotis subflavus*, and *Nycticeius humeralis*) were downloaded from Dryad Digital Repository [41] (http://datadryad.org/). The postman butterfly (*Heliconius melpomene*) genomic sequences were downloaded from Butterfly Genome Database [42] (http://www.butterflygenome.org/). All of the rest of the genome sequences used in this study were downloaded from the National Center for Biotechnology Information.

### Identification of four DNA transposons in *Mengenilla moldrzyki* and other surveyed genomes

Four DNA transposons were identified from the genome of the twisted-wing parasite, *M. moldrzyki*, and they were designated as *Buster1*, *Buster2*, *Spongebob*, and *Kenshin*, respectively. Their consensus sequences were reconstructed using the software DAMBE [43]. Then, their consensus sequences were used as queries to search against Repbase [2] (http://www.girinst.org/) to classify them into known superfamilies. To identify related elements in other species, Blastn [44] searches were performed using nucleotide sequences of the above four DNA transposons query against all GenBank databases and Repbase. Significant hits (>85%) were collected and aligned. Their consensus sequences were also reconstructed and compared among species.

Next, we used these respective consensus sequences to mask each genome to estimate copy number. If one autonomous element and its derivatives coexisted in many studied species genomes (Table 1), their copy numbers were calculated using the following criteria. Fragments that were longer than 600 bp were calculated as copies of autonomous elements as miniature inverted-repeat transposable elements are generally shorter than 600 bp [1]. For MITEs or other non-autonomous elements, all fragments with more than 80% identity and coverage to their consensus sequences were calculated as their copies. Meanwhile, fragments were considered to be a single insertion when they were separated by less than 50 bp. If only one autonomous or non-autonomous element was present in one species, all blast hits with more than 100 bp and 80% identity were used to calculate copy number [26].

### Sequence analysis

ORF of transposons used in this study was predicted using getorf in EMBOSS-6.3.1 package [45]. These elements were aligned using MUSCLE [46]. Shading and minor manual refinements of multiple alignments were deduced using Genedoc [47] and Illustrator CS5. Then, we used the software Bioedit [48] to calculate each pairwise identity of their consensus sequences after all ambiguous and gapped sites were removed. Sequence logos of *Buster1_NA1_SM* and *Buster2_NA2_NH* were created by WebLogo [49] using 30 bp (15 upstream and 15 downstream) flanking their insertion sites.

To determine the relationship of *Buster1* and *Kenshin* with known DNA transposons, transposase sequences of the *hAT* and *piggybac* superfamilies were downloaded from GeneBank and Repbase. Phylogenies were performed with the neighbor-joining method (NJ) using MEGA 4 [50] (pairwise deletion, Poisson correction model, 1,000 bootstrap replicates) based on their transposase sequences. Besides, we also investigated the relationship of *Buster2* from different species. MEGA 4 [50] (pairwise deletion, maximum composite likelihood, 1,000 bootstrap replicates) was used to build phylogenetic trees based on nucleotide sequences of their full-length or nearly full-length copies.

## Additional files

Additional file 1: Table S1. Consensus sequences were used in this study. Sequences multiple alignments were performed using MUSCLE with default parameters. The consensus sequence was generated using DAMBE. Additional file 2: Figure S1. Neighbor-joining phylogenetic tree of genomic copies of *Buster2* from three species, *Mengenilla moldrzyki*, *Rhodnius prolixus*, and *Nycticeius humeralis*. The tree is based on a 230-bp long alignment of 5′ and 3′ termini of genomic copies of *Buster2*. Only bootstrap values >60 are shown. Accession numbers for each element were delineated on each branch. Additional file 3: Figure S2. Insertion bias of *Spongebob_HMa* (A) and paralogous ‘empty’ site of transposons identified in this study (B). Their TSD was shown using rectangles.

## References

[1] Feschotte C, Pritham EJ (2007). DNA transposons and the evolution of eukaryotic genomes. Annu Rev Genet..

[2] Jurka J (2000). Repbase update: a database and an electronic journal of repetitive elements. Trends Genet..

[3] Han MJ, Xu HE, Zhang HH, Feschotte C, Zhang Z (2014). Spy: a new group of eukaryotic DNA transposons without target site duplications. Genome Biol Evol..

[4] Schaack S, Gilbert C, Feschotte C (2010). Promiscuous DNA: horizontal transfer oftransposable elements and why it matters for eukaryotic evolution. Trends Ecol Evol..

[5] Hartl DL, Lohe AR, Lozovskaya ER (1997). Modern thoughts on an ancyent marinere: function, evolution, regulation. Annu Rev Genet..

[6] Roberston HM, Craig NL, Craigie M, Lambowitz A (2002). Evolution of DNA transposons in eukaryotes. Mobile DNA II.

[7] Kidwell MG (1992). Horizontal transfer of *P* elements and other short inverted repeat transposons. Genetica.

[8] Silva JC, Loreto EL, Clark JB (2004). Factors that affect the horizontal transfer of transposable elements. Curr Issues Mol Biol..

[9] Daniels SB, Peterson KR, Strausbaugh LD, Kidwell MG, Chovnick A (1990). Evidence for horizontal transmission of the P transposable element between Drosophila species. Genetics..

[10] Wallau GL, Ortiz MF, Loreto EL (2012). Horizontal transposon transfer in eukarya: detection, bias, and perspectives. Genome Biol Evol..

[11] Capy P, Bazin C, Higuet D, Langin T (1998). Dynamics and evolution of transposable elements (Molecular Biology Intelligence Unit).

[12] Niehuis O, Hartig G, Grath S, Pohl H, Lehmann J, Tafer H (2012). Genomic and morphological evidence converge to resolve the enigma of Strepsiptera. Curr Biol..

[13] Lavoie CA, Platt RN, Novick PA, Counterman BA, Ray DA (2013). Transposable element evolution in Heliconius suggests genome diversity within Lepidoptera. Mob DNA.

[14] Hedges SB, Dudley J, Kumar S (2006). TimeTree: a public knowledge-base of divergence times among organisms. Bioinformatics.

[15] Stadelmann B, Lin LK, Kunz TH, Ruedi M (2007). Molecular phylogeny of New World Myotis (Chiroptera, Vespertilionidae) inferred from mitochondrial and nuclear DNA genes. Mol Phylogenet Evol..

[16] Zhang HH, Shen YH, Xu HE, Liang HY, Han MJ, Zhang Z (2013). A novel hAT element in Bombyx mori and Rhodnius prolixus: its relationship with miniature inverted repeat transposable elements (MITEs) and horizontal transfer. Insect Mol Biol..

[17] Lack JB, Van Den Bussche RA (2010). Identifying the confounding factors in resolving phylogenetic relationships in Vespertilionidae. J Mammal..

[18] Moreno-Vazquez S, Ning J, Meyers BC (2005). hATpin, afamily of MITE-like hAT mobile elements conserved in diverse plant species that forms highly stable secondary structures. Plant Mol Biol..

[19] Tu Z (2000). Molecular and evolutionary analysis of two divergent subfamilies of a novel miniature inverted repeat transposable element in the yellow fever mosquito, Aedes aegypti. Mol Biol Evol..

[20] Arensburger P, Hice RH, Zhou L, Smith RC, Tom AC, Wright JA (2011). Phylogenetic and functional characterization of the hAT transposon superfamily. Genetics.

[21] Datzmann T, von Helversen O, Mayer F (2010). Evolution of nectarivory in phyllostomid bats (Phyllostomidae Gray, 1825, Chiroptera: Mammalia). BMC Evol Biol..

[22] Yang G, Nagel DH, Feschotte C, Hancock CN, Wessler SR (2009). Tuned for transposition: molecular determinants underlying the hyperactivity of a Stowaway MITE. Science..

[23] DeMarco R, Venancio TM, Verjovski-Almeida S (2006). SmTRC1, a novel Schistosoma mansoni DNA transposon, discloses new families of animal and fungi transposons belonging to the CACTA superfamily. BMC Evol Biol..

[24] Volff JN (2006). Turning junk into gold: domestication of transposable elements and the creation of new genes in eukaryotes. Bioessays..

[25] Loreto EL, Carareto CM, Capy P (2008). Revisiting horizontal transfer of transposable elements in Drosophila. Heredity..

[26] Gilbert C, Schaack S, Pace JK, Brindley PJ, Feschotte C (2010). A role for host parasite interactions in the horizontal transfer of transposons across phyla. Nature..

[27] Zhang HH, Xu HE, Shen YH, Han MJ, Zhang Z (2013). The origin and evolution of six miniature inverted-repeat transposable elements in *Bombyx mori* and *Rhodnius prolixus*. Genome Biol Evol..

[28] Novick P, Smith J, Ray D, Boissinot S (2010). Independent and parallel lateral transfer of DNA transposons in tetrapod genomes. Gene.

[29] Lander ES, Linton LM, Birren B, Nusbaum C, Zody MC, Baldwin J (2001). Initial sequencing and analysis of the human genome. Nature.

[30] Waterston RH, Lindblad-Toh K, Birney E, Rogers J, Abril JF, Agarwal P (2002). Initial sequencing and comparative analysis of the mouse genome. Nature.

[31] Pace JK, Feschotte C, Feschotte C (2007). The evolutionary history of human DNA transposons: Evidence for intense activity in the primate lineage. Genome Res..

[32] Ray DA, Pagan HJ, Thompson ML, Stevens RD (2007). Bats with hATs: evidence for recent DNA transposon activity in genus Myotis. Mol Biol Evol..

[33] Ray DA, Feschotte C, Pagan HJ, Smith JD, Pritham EJ, Arensburger P (2008). Multiple waves of recent DNA transposon activity in the bat, Myotis lucifugus. Genome Res..

[34] Pagán HJ, Macas J, Novák P, McCulloch ES, Stevens RD, Ray DA (2012). Survey sequencing reveals elevated DNA transposon activity, novel elements, and variation in repetitive landscapes among vesper bats. Genome Biol Evol..

[35] Thomas J, Schaack S, Pritham EJ (2010). Pervasive horizontal transfer of rolling-circle transposons among animals. Genome Biol Evol..

[36] Simmons NB. Order chiroptera. In: Wilson DE, Reeder DM, editors. Mammal species of the world. 3rd ed. Johns Hopkins University; 2005. p.312-529.

[37] Kathirithamby J (1989). Review of the order Strepsiptera. Syst Ent..

[38] Kathirithamby J (2009). Host-parasitoid associations in Strepsiptera. Annu Rev Ent..

[39] Duan J, Li R, Cheng D, Fan W, Zha X, Cheng T (2010). SilkDB v2.0: a platform for silkworm (Bombyx mori ) genome biology. Nucleic Acids Res.

[40] Lawson D, Arensburger P, Atkinson P, Besansky NJ, Bruggner RV, Butler R (2009). VectorBase: a data resource for invertebrate vector genomics. Nucleic Acids Res.

[41] Pagán HJ, Macas J, Novák P, McCulloch ES, Stevens RD, Ray DA: Data from: Survey sequencing reveals elevated DNA transposon activity, novel elements, and variation in repetitive landscapes among vesper bats. 2012 Dryad Digital Repository. http://dx.doi.org/10.5061/dryad.83164r7v. 10.1093/gbe/evs038 PMC3342881 22491057

[42] Dasmahapatra KK, Walters JR, Briscoe AD, Davey JW, Whibley A, Nadeau NJ (2012). Butterfly genome reveals promiscuous exchange of mimicry adaptations among species. Nature.

[43] Xia X, Xie Z (2001). DAMBE: software package for data analysis in molecular biology and evolution. J Hered..

[44] Altschul SF, Gish W, Miller W, Myers EW, Lipman DJ (1990). Basic local alignment search tool. J Mol Biol..

[45] Rice P, Longden I, Bleasby A (2000). EMBOSS: the European molecular biology open software suite. Trends Genet..

[46] Edgar RC (2004). MUSCLE: multiple sequence alignment with high accuracy and high throughput. Nucleic Acids Res..

[47] Nicholas KB, Nicholas HB, Deerfield DW (1997). GeneDoc: analysis and visualization of genetic variation. EMBNEW News..

[48] Hall TA (1999). BioEdit: a user-friendly biological sequence alignment editor and analysis program for Windows 95/98/NT. Nucleic Acids Symp Ser..

[49] Crooks GE, Hon G, Chandonia JM, Brenner SE (2004). WebLogo: a sequence logo generator. Genome Res..

[50] Tamura K, Dudley J, Nei M, Kumar S (2007). MEGA4: molecular evolutionary genetics analysis (MEGA) software version 4.0. Mol Biol Evol..

