# Supplementary figures and images for: Repeated horizontal transfers of four DNA transposons in invertebrates and bats
Source: Mob DNA. 2015 Jan 17;6:3. doi: 10.1186/s13100-014-0033-1 (PMC4298943; doi:10.1186/s13100-014-0033-1)

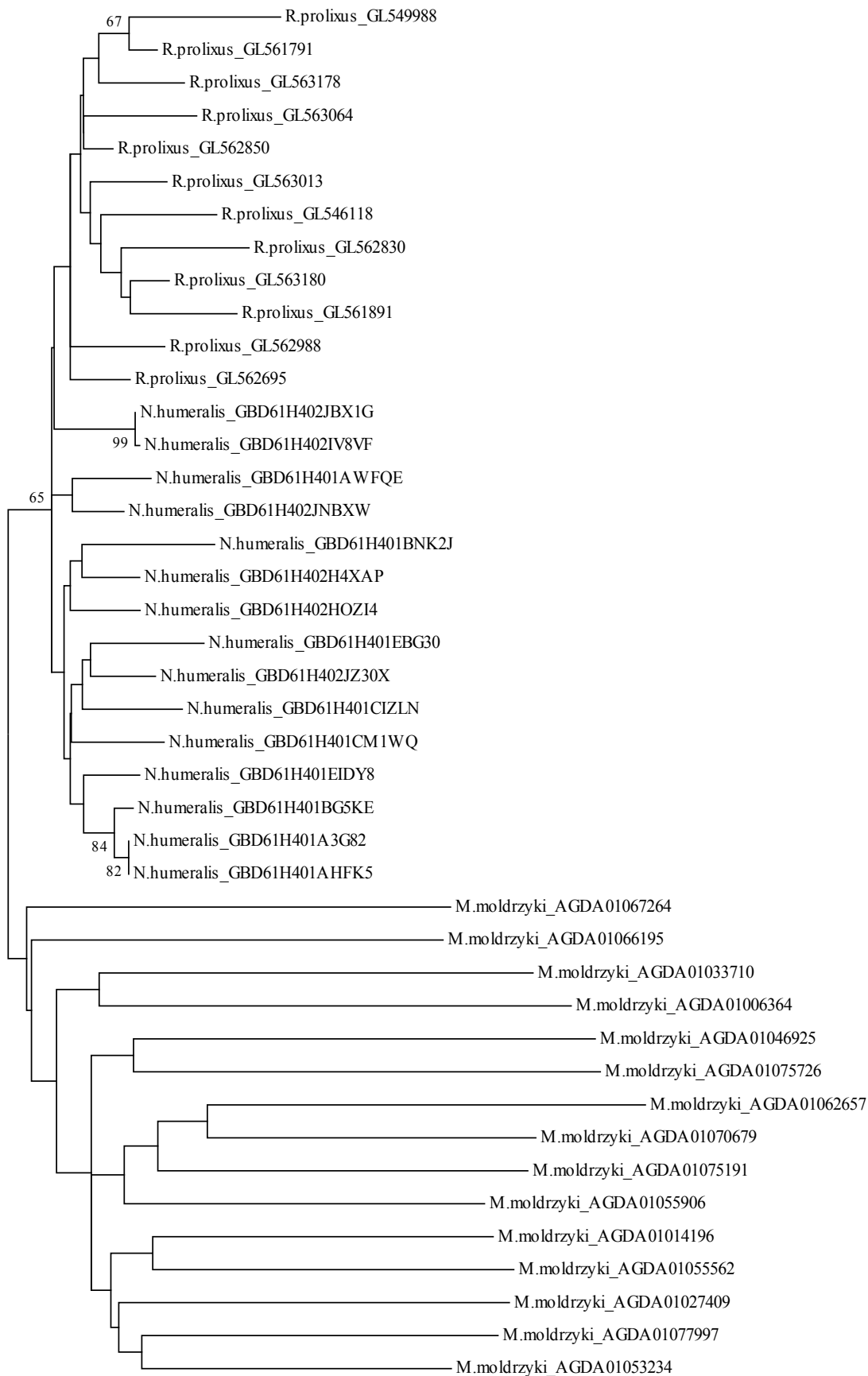

0.01

Supplement: Additional file 2: Figure S1. — Neighbor-joining phylogenetic tree of genomic copies of Buster2 from three species, Mengenilla moldrzyki, Rhodnius prolixus, and Nycticeius humeralis. The tree is based on a 230-bp long alignment of 5′ and 3′ termini of genomic copies of Buster2. Only bootstrap values >60 are shown. Accession numbers for each element were delineated on each branch. [file 13100_2014_33_MOESM2_ESM.pdf]
