# Supplementary material for: Repeated horizontal transfers of four DNA transposons in invertebrates and bats
Source: Mob DNA. 2015 Jan 17;6:3. doi: 10.1186/s13100-014-0033-1 (PMC4298943; doi:10.1186/s13100-014-0033-1)
Supplement: Additional file 3: Figure S2. — Insertion bias of Spongebob_HMa (A) and paralogous ‘empty’ site of transposons identified in this study (B). Their TSD was shown using rectangles. [file 13100_2014_33_MOESM3_ESM.pdf]

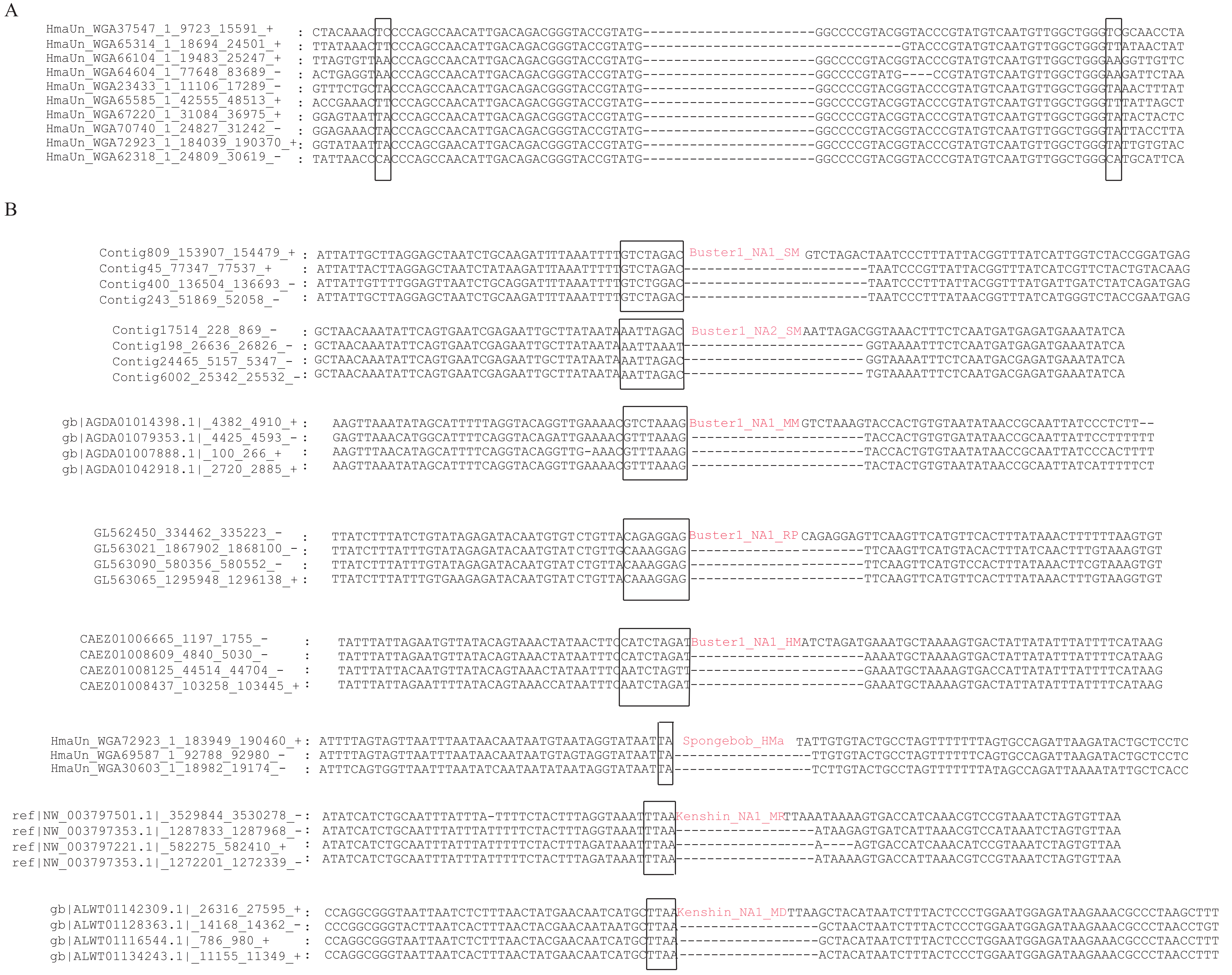
A
HmaUn_WGA37547_1_9723_15591_+ : CTACAAACTCCCCAGCCAACATTGACAGACGGGTACCGTATG----------------------GGCCCCGTACGGTACCCGTATGTCAATGTTGGCTGGGTCGCAACCTA
HmaUn_WGA65314_1_18694_24501_+ : TTATAAACTTCCCAGCCAACATTGACAGACGGGTACCGTATG-----------------------------------GTACCCGTATGTCAATGTTGGCTGGGTTATAACTAT
HmaUn_WGA66104_1_19483_25247_+ : TTAGTGTTAACCCAGCCAACATTGACAGACGGGTACCGTATG----------------------GGCCCCGTACGGTACCCGTATGTCAATGTTGGCTGGGAAGGTTGTTC
HmaUn_WGA64604_1_77648_83689_- : ACTGAGGTAACCCAGCCAACATTGACAGACGGGTACCGTATG----------------------GGCCCCGTATG----CCGTATGTCAATGTTGGCTGGGAAGATTCTAA
HmaUn_WGA23433_1_11106_17289_- : GTTTCTGCTACCCAGCCAACATTGACAGACGGGTACCGTATG----------------------GGCCCCGTACGGTACCCGTATGTCAATGTTGGCTGGGTAAACTTTAT
HmaUn_WGA65585_1_42555_48513_+ : ACCGAAACTTCCCAGCCAACATTGACAGACGGGTACCGTATG----------------------GGCCCCGTACGGTACCCGTATGTCAATGTTGGCTGGGTTTATTAGCT
HmaUn_WGA67220_1_31084_36975_+ : GGAGTAATTACCCAGCCAACATTGACAGACGGGTACCGTATG----------------------GGCCCCGTACGGTACCCGTATGTCAATGTTGGCTGGGTATACTACTC
HmaUn_WGA70740_1_24827_31242_- : GGAGAAACTACCCAGCCAACATTGACAGACGGGTACCGTATG----------------------GGCCCCGTACGGTACCCGTATGTCAATGTTGGCTGGGTATTACCTTA
HmaUn_WGA72923_1_184039_190370_+: GGTATAATTACCCAGCGAACATTGACAGACGGGTACCGTATG----------------------GGCCCCGTACGGTACCCGTATGTCAATGTTGGCTGGGTATTGTGTAC
HmaUn_WGA62318_1_24809_30619_- : TATTAACCCACCCAGCCAACATTGACAGACGGGTACCGTATG----------------------GGCCCCGTACGGTACCCGTATGTCAATGTTGGCTGGGCATGCATTCA
B
Contig809_153907_154479_+ : ATTATTGCTTAGGAGCTAATCTGCAAGATTTAAATTTTGTCTAGAC Buster1_NA1_SM GTCTAGACTAATCCCTTTATTACGGTTTATCATTGGTCTACCGGATGAG
Contig45_77347_77537_+ : ATTATTACTTAGGAGCTAATCTATAAGATTTAAATTTTGTCTAGAC------------------------------TAATCCCGTTATTACGGTTTATCATCGTTCTACTGTACAAG
Contig400_136504_136693_- : ATTATTGTTTTGGAGTTAATCTGCAGGATTTAAATTTTGTCTGGAC------------------------------TAATCCCTTTATTACGGTTTATGATTGATCTATCAGATGAG
Contig243_51869_52058_- : ATTATTGCTTAGGAGCTAATCTGCAAGATTTAAATTTTGTCTAGAC------------------------------TAATCCCTTTATAACGGTTTATCATGGGTCTACCGAATGAG
Contig17514_228_869_- : GCTAACAAATATTCAGTGAATCGAGAATTGCTTATAATAAATTAGAC Buster1_NA2_SM AATTAGACGGTAAACTTTCTCAATGATGAGATGAAATATCA
Contig198_26636_26826_- : GCTAACAAATATTCAGTGAATCGAGAATTGCTTATAATAAATTAAAT------------------------ GGTAAAATTTCTCAATGATGAGATGAAATATCA
Contig24465_5157_5347_- : GCTAACAAATATTCAGTGAATCGAGAATTGCTTATAATAAATTAGAC------------------------ GGTAAAATTTCTCAATGACGAGATGAAATATCA
Contig6002_25342_25532_-: GCTAACAAATATTCAGTGAATCGAGAATTGCTTATAATAAATTAGAC------------------------ TGTAAAATTTCTCAATGACGAGATGAAATATCA
gb|AGDA01014398.1|_4382_4910_+ : AAGTTAAATATAGCATTTTTAGGTACAGGTTGAAAACGTCTAAAG Buster1_NA1_MM GTCTAAAGTACCACTGTGTAATATAACCGCAATTATCCCTCTT--
gb|AGDA01079353.1|_4425_4593_- : GAGTTAAACATGGCATTTTCAGGTACAGATTGAAAACGTTTAAAG ------------------------TACCACTGTGTGATATAACCGCAATTATTCCTTTTTT
gb|AGDA01007888.1|_100_266_+ : AAGTTTAACATAGCATTTTCAGGTACAGGTTG-AAACGTTTAAAG ------------------------TACCACTGTGTAATATAACCGCAATTATCCCACTTTT
gb|AGDA01042918.1|_2720_2885_+ : AAGTTAAATATAGCATTTTCAGGTACAGGTTGAAAACGTTTAAAG ------------------------TACTACTGTGTAATATAACCGCAATTATCATTTTTCT
GL562450_334462_335223_- : TTATCTTTATCTGTATAGAGATACAATGTGTCTGTTACAGAGGAG Buster1_NA1_RP CAGAGGAGTTCAAGTTCATGTTCACTTTATAAACTTTTTTAAGTGT
GL563021_1867902_1868100_- : TTATCTTTATTTGTATAGAGATACAATGTATCTGTTGCAAAGGAG ------------------------TTCAAGTTCATGTACACTTTATCAACTTTGTAAAGTGT
GL563090_580356_580552_- : TTATCTTTATTTGTATAGAGATACAATGTATCTGTTACAAAGGAG ------------------------TTCAAGTTCATGTCCACTTTATAAACTTCGTAAAGTGT
GL563065_1295948_1296138_+ : TTATCTTTATTTGTGAAGAGATACAATGTATCTGTTACAAAGGAG ------------------------TTCAAGTTCATGTTCACTTTATAAACTTTGTAAGGTGT
CAEZ01006665_1197_1755_- : TATTTATTAGAATGTTATACAGTAAACTATAACTTCCATCTAGAT Buster1_NA1_HM ATCTAGATGAAATGCTAAAAGTGACTATTATATTTATTTTCATAAG
CAEZ01008609_4840_5030_- : TATTTATTAGAATGTTATACAGTAAACTATAATTTCCATCTAGAT------------------------AAAATGCTAAAAGTGACTATTATATTTATTTTCATAAG
CAEZ01008125_44514_44704_- : TATTTATTACAATGTTATACAGTAAACTATAATTTCAATCTAGTT------------------------GAAATGCTAAAAGTGACCATTATATTTATTTTCATAAG
CAEZ01008437_103258_103445_+ : TATTTATTAGAATTTTATACAGTAAACCATAATTTCAATCTAGAT------------------------GAAATGCTAAAAGTGACTATTATATTTATTTTCATAAG
HmaUn_WGA72923_1_183949_190460_+ : ATTTTAGTAGTTAATTTAATAACAATAATGTAATAGGTATAATTA Spongebob_HMa TATTGTGTACTGCCTAGTTTTTTTAGTGCCAGATTAAGATACTGCTCCTC
HmaUn_WGA69587_1_92788_92980_- : ATTTTAGTAGTTAATTTAATAACAATAATGTAGTAGGTATAATTA-------------------TTGTGTACTGCCTAGTTTTTCAGTGCCAGATTAAGATACTGCTCCTC
HmaUn_WGA30603_1_18982_19174_- : ATTTCAGTGGTTAATTTAATATCAATAATATAATAGGTATAATTA-------------------TCTTGTACTGCCTAGTTTTTTTATAGCCAGATTAAAATATTGCTCACC
ref|NW_003797501.1|_3529844_3530278_-: ATATCATCTGCAATTTATTTA-TTTTCTACTTTAGGTAAATTTAA Kenshin_NA1_MR TTAAATAAAAGTGACCATCAAACGTCCGTAAATCTAGTGTTAA
ref|NW_003797353.1|_1287833_1287968_-: ATATCATCTGCAATTTATTTATTTTTCTACTTTAGGTAAATTTAA-------------------ATAAGAGTGATCATTAAACGTCCATAAATCTAGTGTTAA
ref|NW_003797221.1|_582275_582410_+ : ATATCATCTGCAATTTATTTATTTTTCTACTTTAGATAAATTTAA-------------------A----AGTGACCATCAAACATCCGTAAATCTAGTGTTAA
ref|NW_003797353.1|_1272201_1272339_-: ATATCATCTGCAATTTATTTATTTTTCTACTTTAGATAAATTTAA-------------------ATAAAAGTGACCATTAAACGTCCGTAAATCTAGTGTTAA
gb|ALWT01142309.1|_26316_27595_+ : CCAGGCGGGTAATTAATCTCTTTAACTATGAACAATCATGCTTAA Kenshin_NA1_MD TTAAGCTACATAATCTTTACTCCCTGGAATGGAGATAAGAAACGCCCTAAGCTTT
gb|ALWT01128363.1|_14168_14362_- : CCCGGCGGGTACTTAATCACTTTAACTACGAACAATAATGCTTAA-------------------GCTAACTAATCTTTACTCCCTGGAATGGAGATAAGAAACGCCCTAACCTGT
gb|ALWT01116544.1|_786_980_+ : CCAGGCGGGTAATTAATCTCTTTAACTACGAACAATCATGCTTAA-------------------GCTACATAATCTTTACTCCCTGGAATGGAGATAAGAAACGCCCTAACCTTT
gb|ALWT01134243.1|_11155_11349_+ : CCAGGCGGGTAATTAATCACTTTAACTATGAACAATCATGCTTAA-------------------ACTACATAATCTTTACTCCCTGGAATGGAGATAAGAAACGCCCTAACCTTT
